# Minimal change disease following COVID-19 vaccination: A systematic review

**DOI:** 10.1371/journal.pone.0297568

**Published:** 2024-03-05

**Authors:** Konstantinos S. Kechagias, Joshua D. Laleye, Jan Drmota, Georgios Geropoulos, Georgios Kyrtsonis, Marina Zafeiri, Konstantinos Katsikas Triantafyllidis, Dimitra Stathi

**Affiliations:** 1 Department of Metabolism, Digestion and Reproduction, Faculty of Medicine, Imperial College London, London, United Kingdom; 2 Western General Hospital, Lothian NHS, Edinburgh, United Kingdom; 3 School of Cardiovascular Medicine and Sciences, King’s College London, London, United Kingdom; 4 Department of Nutrition and Dietetics, Royal Marsden NHS Foundation Trust, London, United Kingdom; 5 Department of Endocrinology and Diabetes, King’s College Hospital NHS Foundation Trust, London, United Kingdom; University of KwaZulu-Natal, SOUTH AFRICA

## Abstract

**Background:**

The newly developed COVID-19 vaccines are highly effective and safe. However, a small portion of vaccine recipients experience a wide range of adverse events. Recently, glomerular disease, including the development of Minimal Change Disease (MCD), has been observed after administration of different COVID-19 vaccines, although causality remains a matter of debate.

**Aim:**

The aim of this systematic review was to comprehensively examine the available literature and provide an overview of reported cases of MCD following vaccination against SARS-CoV-2.

**Results:**

We identified 46 eligible articles which included 94 cases with MCD following COVID-19 vaccination of which one case was reported twice due to a second relapse. Fifty-five participants were males (59.1%, 55/93) and 38 (40.9%, 38/93) were females with a mean age of 45.02 years (SD:20.95). From the included patients 50 (50/94, 53.1%) were described as new-onset and 44 (46.9%, 44/94) as relapse. On average, symptomatology developed 16.68 days (SD: 22.85) after the administration of the vaccine irrespective of the dose. Data about symptoms was reported in 68 cases with the most common being oedema (80.8%, 55/68), followed by weight gain (26.5%, 18/68) and hypertension (16.1%, 11/68). In terms of outcome, more than half of the patients went into remission (61%, 57/94), while 18 recovered or improved post treatment (19.1%, 18/94). Two people relapsed after treatment (2.1%, 2/94) and two cases (2.1%, 2/94) were reported as not recovered.

**Conclusion:**

MCD is possibly a condition clinicians may see in patients receiving COVID-19 vaccines. Although this adverse event is uncommon, considering the limited published data and the absence of confirmed causality, increased clinical awareness is crucial for the early recognition and optimal management of these patients.

## Introduction

In late 2019, a global pandemic, which created extraordinary socio-economic consequences, emerged due to an outbreak of an uncommon viral pneumonia [1–3]. The aetiological factor was later identified as a previously unknown strain of coronavirus named Severe Acute Respiratory Syndrome Coronavirus 2 (SARS-CoV-2), responsible for the onset of coronavirus disease 2019 (COVID-19). The disease has since spread extensively, impacting hundreds of millions of individuals across the globe [4, 5].

Various vaccines have been utilised successfully against SARS-CoV-2 such as COMIRNATY (BioNTech-Pfizer’s COVID-19 mRNA vaccine BNT162b2), COVID-19 Vaccine Moderna (Moderna’s mRNA vaccine-1273), VAXZEVRIA (AstraZeneca-Oxford University’s ChAdOx1-nCoV19), COVID-19 Vaccine Janssen (Janssen’s Ad26.COV2.S) and CoronaVac COVID19 vaccine (Sinovac Biotech’s Vero cell) [6, 7]. Currently, nearly two-thirds of the global population have received at least one dose of a COVID-19 vaccine, with more than 13 billion doses administered worldwide [8].

A plethora of published studies have demonstrated the safety and effectiveness of the aforementioned vaccines, with only infrequent adverse events reported in the literature [9–12]. Nonetheless, isolated adverse reactions after COVID-19 vaccine administration are unavoidable, given the vast amount of vaccination doses needed to curb the spread of COVID-19 [13, 14]. At present, patients commonly experience various reported adverse symptoms, such as muscle pain, fever, headache, nausea, and vomiting. In addition to the frequently observed adverse effects following COVID-19 vaccination, patients have also reported a wide range of complaints and symptoms, including immune-mediated adverse events [13, 15–18].

Recently, there is a growing number of reports regarding the development of Minimal Change Disease (MCD) in patients following their initial or second COVID-19 vaccine doses; However, these cases have not yet undergone thorough investigation, and the administration of COVID-19 vaccines has not been recognised as a causative factor for renal dysfunction. To address this gap, our study systematically analysed the existing literature to present a comprehensive summary of documented cases of MCD following SARS-CoV-2 vaccination.

## Methods

This review was reported based on the “Preferred Reporting Items for Systematic Reviews and Meta-Analyses” (PRISMA) guidelines (S1 Fig).

### Literature search

Two reviewers (KSK, JDL) searched PubMed and Scopus library databases from inception until January 2023 independently. The search included the following terms: “(COVID 19 vaccin* OR SARS-COV2 vaccin*) AND (minimal change disease OR glomerulonephritis OR nephrotic OR nephritic)”. There were no limitations placed regarding study design, geographic region, or language. Additionally, a manual search of references cited in the included articles and relevant published reviews was conducted to identify any missed studies. Discrepancies during the literature search were resolved by a third investigator (DS).

### Eligibility criteria

We included studies that provided data for new onset or relapse of MCD following COVID-19 vaccination with at least one dose. All study designs were considered eligible for inclusion. Review articles, abstracts submitted in conferences and non-peer reviewed sources were not eligible for inclusion. Studies on in vitro and animal models were excluded.

### Data extraction and handling

In all studies, patient data was retrieved and handled by two authors (JDL, JD) who conducted the data extraction independently. We collected the following information: sex, age, comorbidities, vaccine type, number of doses received, presenting complains and symptoms, history of previous COVID-19 infection, laboratory tests including antibodies, primary diagnosis, imaging findings, therapeutic management and clinical outcome. Any disagreements were discussed and resolved by a third author (KSK).

### Quality assessment

The quality of the included studies was assessed using the criteria established by the Task Force for Reporting Adverse Events of the International Society for Pharmacoepidemiology (ISPE) and the International Society of Pharmacovigilance (ISoP) [19]. The evaluation was based on the satisfactory reporting of 12 different elements, including the title, patient demographics, current health status, medical history, physical examination, patient disposition, drug identification, dosage, administration/drug reaction interface, concomitant therapies, adverse events, and discussion. Each element was assigned a score of either 0 (lack of information) or 1 (information present) for the studies.

## Results

### Study characteristics

**Fig 1:**
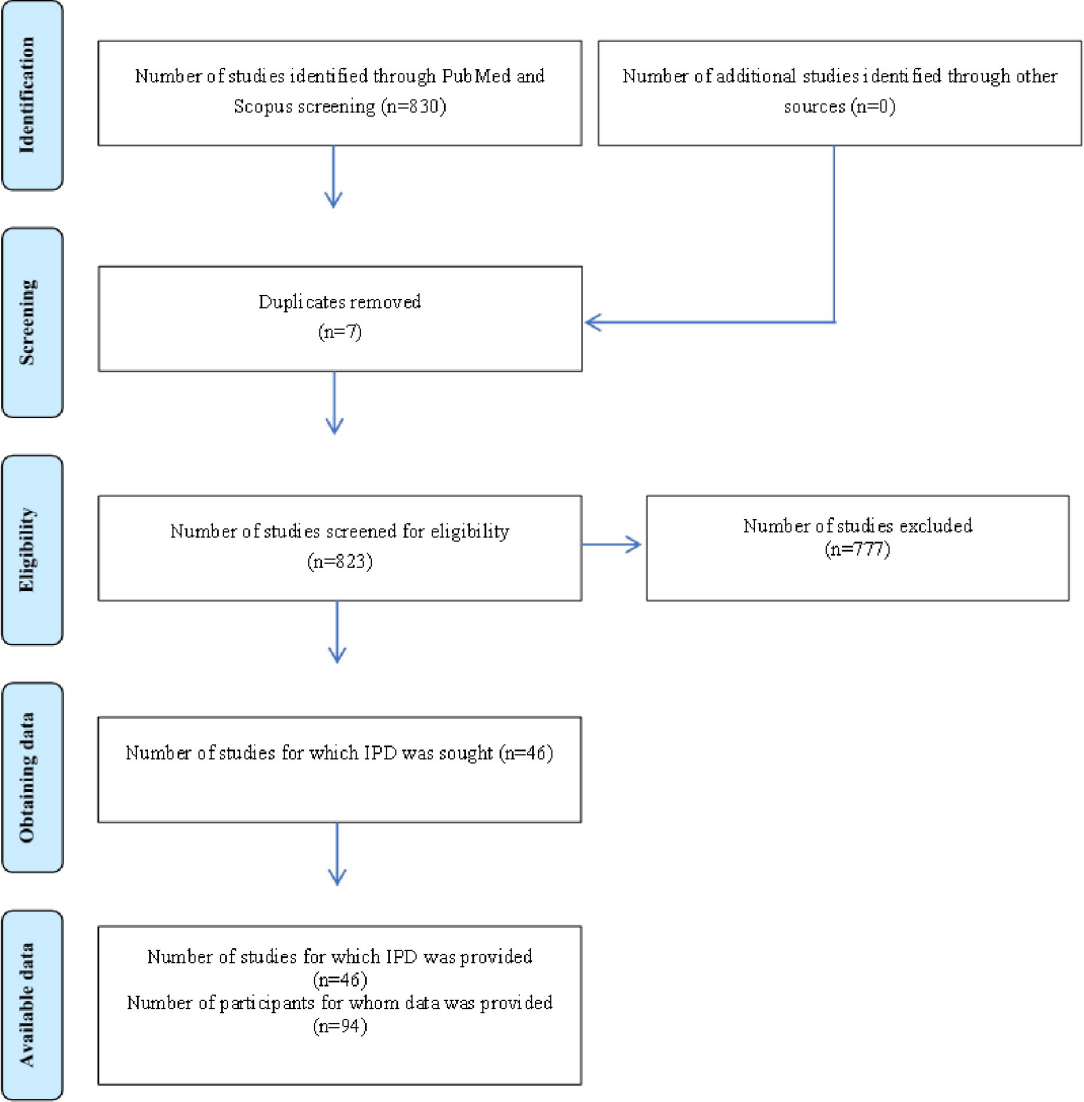
PRISMA flowchart. IPD: individual patient data.

The initial literature search yielded 830 publications. In the first screening 777 studies were excluded as irrelevant. Forty-six studies [20–65] were found to be eligible for the systematic review based on the predefined inclusion criteria (Fig 1). Twenty of the studies were conducted in Asia, 16 in Europe, 9 in Americas, and 1 in Australia. Seven studies were case series and 39 were case reports (Table 1).

**Table 1:** Characteristics of the included studies.

Author, Year, Country	Case number, Age, Gender	Comorbidities	Previous glomerulopathy	Previous COVID-19 infection	COVID-19 vaccine type and dose	New onset/relapse of minimal change disease post vaccination	Main presenting symptoms	Days for the onset of symptoms	Treatment	Outcome
Marampudi 2022 USA	case 1 54 F	Hypertension	MCD	none	mRNA (Pfizer), first	relapse	• Lower Limb Oedema • Foamy Urine	7	Prednisolone (50mg/day)	prednisone taper rituximab if relapses
Pella 2022 Greece	case 1 18 M	None	None	none	mRNA (Pfizer), first	new	• Nausea • Bloating • Abdominal Pain • Lower Limb Oedema • Weight Gain	11	Irbesartan 150 mg Methylprednisolone 48 mg	Complete remission in 6 weeks
Alhosaini 2022 UAE	case 1 16 M	None	n/a	NA	mRNA (Pfizer), second	new	• Lower Limb Oedema • Ankle Swelling • Abdominal Pain • Weight gain	7	Prednisone 60mg, furosemide, Olmesartan	Oedema resolved after 1 week
Mochizuki 2022 Japan	case 1 25 F	None	None	NA	mRNA (Moderna), first	new	• Facial Oedema • Peripheral oedema • Weight gain	26	IV Methylprednisolone 500 mg/day for 3 days Oral Prednisolone 45mg/day.	Complete remission by day 10
Park 2022 Korea	case 1 34 M	NA	None	NA	mRNA (Moderna), second	new	• Peri-ocular oedema • Dyspnoea • Weight gain	3	Prednisolone	Remission after 11 weeks
	case 2 60 M	NA	None	NA	mRNA (Moderna), second	new	• Oedema • Weight gain	5	Steroids	Complete remission after 2 weeks
Hartley 2022 UK	case 1 80s F	None	None	NA	mRNA (Pfizer), first	new	• Oedema • Reduced urine output • Hypertension	2	Loop diuretics, Low molecular weight heparin, Steroids, Levothyroxine	Complete remission
	case 2 40s M	Wolf-Parkinson-White Syndrome, Cardiac ablation	MCD	NA	mRNA (Pfizer), first	relapse	• Oedema • Diarrhoea • Vomiting	1	Furosemide, Prednisolone, Ciclosporin	Complete remission
Leong 2021 Singapore	case 1 42 F	None	MCD	NA	mRNA (Moderna), second	relapse	• Oedema • Frothy urine	11	Prednisolone	Remission within 2 weeks
	case 2 30 M	None	MCD	NA	mRNA (Pfizer), second	relapse	• Oedema • Frothy urine	7	Prednisolone	Remission within 2 weeks
Tanaka 2022 Japan	case 1 69 F	Hypertension, Hyperlipidaemia	None	NA	mRNA (Pfizer), second	new	• Oedema • Weight gain	9–18 days	Prednisolone	Complete remission within 1 month
Jongvilaikasem 2022 Thailand	case 1 14 M	None	None	NA	mRNA (Pfizer), first	new	• Oedema • Hypertension	5	Corticosteroids	Partial remission after 5 weeks treatment
Marinaki 2021 Greece	case 1 55 F	Hypothyroidism	None	NA	mRNA (Pfizer), first and second	mode (after second dose)	• Oedema • Weight gain	4 days after first dose. 1 day after second	Prednisolone	Remission after 10 days
Biradar 2021 India	case 1 22 M	None	None	NA	Viral Vector (Oxford-Astrazeneca), first	new	• Oedema	11	Prednisolone	Remission after 1 week
Unver 2021 Turkey	case 1 67 F	Type 2 diabetes mellitus	None	NA	Inactivated (Sinovac), first	new	• Oedema • Weight gain	20	Ramipril, Valsartan, Nebivolol, Rosuvastatin, Furosemide	Represented after second dose
Lebedev 2021 Israel	case 1 50 M	None	None	NA	mRNA (Pfizer), first	new	• Oedema • Abdominal distension	4 days	Prednisolone	Improved 17 days later
Hanna 2021 Canada	case 1 60 M	None	None	NA	mRNA (Pfizer), first	new	• Oedema • Fatigue • Shortness of breath on exertion	10	Ramipril, Furosemide, Prednisolone	Remission from 14 days confirmed 3 weeks later
Baskaran 2022 Australia	case 1 31 F	NA	None	NA	mRNA (Pfizer), second	new	• Oedema	21	High-dose steroids	Good response to treatment
	case 2 55 M	NA	None	NA	Viral Vector (Oxford-Astrazeneca), second	new	• Oedema • Ascites	7	Prednisolone	Improved kidney function and proteinuria
Thappy 2021 Qatar	case 1 43 M	None	None	none	mRNA (Moderna), first	new	• Oedema • Dyspnoea	7	Furosemide, Amlodipine, Prednisolone	No oedema, raised serum albumin, reduced urine protein after 2 weeks
Abdulgayoom 2021 Qatar	case 1 45 F	Hypothyroidism, Atopic dermatitis, Heterozygous factor V mutation	None	NA	mRNA (Pfizer), first	new	• Oedema • Abdominal distention • Foamy urine • Abdominal ascites	4	Furosemide, Prednisolone, Vitamin D, Calcium, Pantoprazole, Trimethoprim/Sulfamethoxazole	NA
Klomjit 2021 USA	case 1 83 M	NA	None	NA	mRNA (Moderna), second	new	• AKI	28	High dose steroids	Responded to treatment at 1 month follow-up
	case 2 67 F	NA	NA	NA	mRNA (Moderna), second	relapse	• Oedema	21	High dose steroids, Rituximab	Responded to treatment at 2-month follow-up
Lim 2021 Korea	case 1 51 M	None	None	NA	Viral Vector (Janssen), first	new	• Oedema • Reduced urination • Weight gain	7	Furosemide, Methylprednisolone	Decreased serum creatinine, increased serum albumin after 7 days
Salem 2021 USA	case 1 33 F	None	MCD	NA	mRNA (Moderna), second	relapse	• Oedema • Headache • Vomiting • Hypertension	21	NA	NA
	case 2 41 F	Asthma	None	NA	mRNA (Pfizer), second	new	• Fever • Oedema • Weight gain • Hypertension	5	NA	NA
	case 3 34 F	None	MCD	NA	mRNA (Pfizer), second	relapse	• Oedema • Abdominal pain	28	NA	NA
Morlidge 2021 UK	case 1 30 M	None	MCD	NA	Viral Vector (Oxford-Astrazeneca), first	relapse	• Headache • Frothy urine	2	Prednisolone	Complete remission after 10 days treatment
	case 2 40 F	None	MCD	NA	Viral Vector (Oxford-Astrazeneca), first	relapse	• Headache • Frothy urine • Oedema	1	Prednisolone increased	Complete remission within 2 weeks
Özkan 2022 Turkey	case 1 33 F	None	MCD	NA	inactive SARS-CoV-2, second	relapse	• Foamy urine • Oedema	14	Methylprednisolone	NA
Kervella 2021 France	case 1 34 F	None	MCD	NA	mRNA (Pfizer), first	relapse	• Oedema	10	Increased corticosteroid dose	Complete remission after second dose relapse
Chandra 2022 USA	case 1 23 F	None	None	NA	mRNA (Moderna), second	new	• Oedema • Elevated blood pressure	7	Corticosteroids	Complete remission after 4 weeks
	case 2 74 M	Hypertension	None	NA	mRNA (Pfizer), second	new	• Oedema • Weight gain	2	Supportive therapy	Complete remission after ~6 weeks
	case 3 72 F	Hypertension, Obesity, Dyslipidaemia	None	None	Viral Vector (Oxford-AstraZeneca), First	new	• Oedema • Dyspnoea • Fatigue	14	Prednisolone	Complete remission of proteinuria and improved creatinine and albumin after 2 weeks treatment
	case 4 71 M	Acute myeloid leukaemia, Allogeneic hematopoietic stem cell transplantation, Glucocorticoid-induced diabetes, Mild GVHD in liver	MCD (GVHD)	NA	mRNA (Moderna), second	relapse	• Foamy and dark urine • Oedema • Abdominal bloating	7	Prednisolone, Rituximab, Loop diuretic	Complete remission after 7 months
Hummel 2022 France	case 1 38 M	NA	NA	NA	Viral Vector (Oxford-AstraZeneca), first	relapse	NA	14	Corticosteroids, Mycophenolate Mofetil	Complete remission after 1 month
	case 3 74 M	NA	NA	NA	mRNA (Pfizer), first	relapse	NA	21	Corticosteroids, Calcineurin inhibitor	Complete remission after 3 months
	case 4 46 F	NA	NA	NA	mRNA (Pfizer), first	relapse	NA	11	Corticosteroids, Calcineurin inhibitor	Complete remission after 1 month
	case 5 23 M	NA	NA	NA	mRNA (Pfizer), first	relapse	NA	21	Corticosteroids, Obinutuzumab	Complete remission after 1 month
	case 6 30 F	NA	NA	NA	mRNA (Pfizer), second	relapse	NA	6	Corticosteroids, Rituximab	Complete remission after 1 month
	case 7 36 F	NA	NA	NA	mRNA (Pfizer), first	relapse	NA	10	Corticosteroids, Rituximab	Complete remission after 1 month
	case 8 41 F	NA	NA	NA	mRNA (Pfizer), first	relapse	NA	30	Corticosteroids, Calcineurin inhibitor	Complete remission after 1 month
	case 9 16 M	NA	NA	NA	mRNA (Pfizer), first	relapse	NA	15	Corticosteroids	Complete remission after 1 month
	case 10 19 M	NA	NA	NA	mRNA (Pfizer), first	relapse	NA	21	Corticosteroids	Complete remission after 1 month
	case 11 48 M	NA	NA	NA	mRNA (Moderna), first	relapse	NA	7	Corticosteroids, Mycophenolate mofetil	Complete remission after 1 month
	case 12 40 M	NA	NA	NA	mRNA (Pfizer), first	relapse	NA	7	Corticosteroids	Complete remission after 1 month
	case 14 83 M	NA	NA	NA	Viral Vector (Oxford-AstraZenecca), second	relapse	NA	20	Corticosteroids	Complete remission after 3 months
	case 15 53 F	NA	NA	NA	mRNA (Pfizer), first	relapse	NA	26	Corticosteroids	Complete remission after 1 month
	case 16 25 M	NA	NA	NA	mRNA (Pfizer), first	relapse	NA8	21	Corticosteroids, Mycophenolate mofetil	Complete remission after 1 month
	case 17 19 M	NA	NA	NA	mRNA (Pfizer), second	relapse	NA	25	Corticosteroids	Complete remission after 1 month
	case 18 15 M	NA	NA	NA	mRNA (Pfizer), first	relapse	NA	28	Corticosteroids	Complete remission after 1 month
	case 19 31 M	NA	NA	NA	mRNA (Pfizer), first	relapse	NA	21	Corticosteroids	Complete remission after 1 month
	case 20 21 M	NA	NA	NA	mRNA (Pfizer), second	relapse	NA	20	Corticosteroids	Complete remission after 3 months
	case 21 42 M	NA	NA	NA	Viral Vector (Oxford-AstraZeneca), first	relapse	NA	11	Corticosteroids	Complete remission after 3 months
	case 22 72 M	NA	NA	NA	mRNA (Pfizer), third	relapse	NA	7	Corticosteroids, Mycophenolate mofetil	NA
	case 23 18 F	NA	NA	NA	mRNA (Pfizer), first	relapse	NA	14	Corticosteroids, Mycophenolate mofetil	Complete remission after 1 month
	case 24 16 F	NA	NA	NA	mRNA (Moderna), second	relapse	NA	1	Corticosteroids	Complete remission after 1 month
	case 25 72 M	NA	NA	NA	mRNA (Pfizer), third	relapse	NA	2	Corticosteroids	NA
Güngör 2022 Turkey	case 1 17 F	No	Idiopathic nephrotic syndrome	NA	modRNA, second	relapse	• Oedema	19	Corticosteroids	Remission after 2 weeks of treatment
	case 2 17.5 F	No	Idiopathic nephrotic syndrome	NA	NA, second	relapse	• Oedema	12	Corticosteroids	Remission after 2 weeks of treatment
Fenoglio 2022 Italy	case 5 36 M	NA	No	NA	mRNA (Pfizer), second	new	• Urinary abnormalities	82	Rituximab	NA
	case 7 82 M	NA	No	NA	mRNA (Moderna), second	new	• Renal failure • Nephrotic syndrome	79	Glucocorticoids	NA
	case 8 54 F	NA	No	NA	mRNA (Moderna), second	new	• Nephrotic syndrome	62	Glucocorticoids	NA
	case 12 42 F	NA	No	NA	mRNA (Pfizer), second	new	• Renal failure • Nephrotic syndrome	88	MC	NA
	case 16 20 M	NA	No	NA	mRNA (Pfizer), first	new	• Nephrotic syndrome	46	Rituximab	NA
Lim 2022 Korea	case 2 52 M	No	No	NA	Viral Vector (Janssen), first	new	• Oedema • Nephrotic syndrome • Weight gain	7	Prednisolone	Complete response at 31 weeks
Dormann 2021 Germany	case 1 78 M	Arterial hypertension, Coronary heart disease, Hyperlipoproteinemia, COPD, Allergies	No	none	mRNA (Pfizer), first	new	• Oedema • Weight gain	4	Diuretics	Relapse after second dose *(see row below)*
	case 1 (2) 78 M	*(See row above)*	*(See row above)*	*(See row above) *	second	relapse	• Oedema • Weight gain • Pleural effusion	14	Prednisolone, Diuretics, Anticoagulants	Partial remission, reduced proteinuria and weight loss after 3 weeks
	case 2 31 F	Lipedema	No	none	Viral Vector (Janssen), first	new	• Oedema • Foamy urine • Syncope with orthostatic dysregulation	0	Prednisolone, Antibiotics, Immunoglobulin, Rituximab, Anticoagulation, Diuretics	Complete remission with mild hyperlipoproteinemia at day 52
Anupama 2021 India	case 1 19 F	NA	No	NA	Viral Vector (Oxford-AstraZeneca), first	new	• Oedema	8	Prednisolone	Clinical and biochemical remission
Schwotzer 2021 Switzerland	case 1 22 M	No	MCD	NA	mRNA (Pfizer), NA	relapse	• Chills and low-grade fever • Proteinuria	2	Prednisolone, Tacrolimus	Remission after 17 days treatment
Hong 2022 Taiwan	case 1 51 M	No	No	NA	mRNA (Moderna), second	new	• Oedema • Foamy urine	3	Prednisolone, Angiotensin 2 receptor blocker	Complete remission at 10 weeks treatment
Timmermans 2022 Netherlands	case 1 64 F	NA	No	none	Viral Vector (Oxford-AstraZeneca), first	new	• Oedema	7	Prednisolone	Complete remission after 4 months
	case 2 34 M	NA	No	none	mRNA (Pfizer), second	new	NA	28	No	NA
	case 3 74 M	NA	No	none	mRNA (Pfizer), second	new	NA	42	Prednisolone	NA
Nakazawa 2022 Japan	case 1 15 M	No	No	yes	mRNA (Pfizer), first	new	• Oedema • Weight gain	4	Prednisolone	Complete remission at 12 days of treatment
Arias 2022 Spain	case 1 28 F	No	Idiopathic nephrotic syndrome	yes	Viral Vector (Oxford-AstraZeneca), first	relapse	• Oedema	2	Prednisolone, Atorvastatin, Antiplatelet therapy, Omeprazole, Trimethoprim-sulfamethoxazole	Negative proteinuria and no oedema after 4 weeks of treatment
Haider 2022 Italy	case 1 63 M	No	MCD	NA	mRNA (Pfizer), booster	relapse	• Oedema • Weight gain • Elevated blood pressure	< 7	Prednisolone	Normal protein:creatinie ratio after 2 weeks treatment
Fehr 2021 Switzerland	case 1 65 M	Collagenous colitis	No	NA	mRNA (Moderna), first	new	• Nephrotic syndrome • AKI	8	Dialysis, Immunosuppressive therapy	Complete remission after treatment
Nagai 2022 Japan	case 1 22 M	No	No	NA	mRNA (Pfizer), first	new	• Oedema	9	Heparin, Prednisolone, Furosemide	Clinical signs disappeared on 7^th^ day of treatment
Caza 2021 USA	case 3 70 F	NA	No	none	mRNA (Pfizer), second	new	• AKI • Nephrotic syndrome	< 7	Steroid therapy	Recovery at 4 weeks
	case 4 43 F	NA	No	none	mRNA (Pfizer), second	new	• Nephrotic syndrome	14	Steroid therapy	Recovery at 4 weeks
	case 5 79 M	NA	No	none	mRNA (NA), first	new	• AKI • Nephrotic syndrome	< 14	Steroid therapy	Recovery at 4 weeks
	case 6 72 M	NA	No	none	mRNA (Moderna), second	new	Nephrotic syndrome	7	Steroid therapy, ACEi	Recovery at 2 weeks
	case 7 47 F	NA	No	none	mRNA (Pfizer), second	new	• AKI • Nephrotic syndrome	< 14	Dialysis, Steroid therapy, ACEi	No recovery at 4 weeks
	case 8 23 M	NA	No	none	Viral Vector (Oxford-AstraZeneca), first	new	• AKI • Nephrotic syndrome	14	Steroid therapy, Dialysis	Recovery at 3 weeks
	case 9 45 F	NA	No	none	mRNA (Moderna), first	new	• Nephrotic syndrome	< 14	Steroid therapy	NA
Fornara 2022 Italy	case 4 66 F	Hypertension TIA	No	NA	mRNA (Pfizer), second	new	NA	160	Oral steroids	Partial remission after 56 days
Leclerc 2021 Canada	case 1 71 M	Dyslipidaemia	No	NA	Viral Vector (Oxford-AstraZeneca), first	new	• Oedema • Elevated blood pressure • AKI	1	Methylprednisolone, Prednisolone, Haemodialysis	Improvement after 30 days treatment
Mancianti 2021 Italy	case 1 39 M	No	MCD	none in the weeks prior	mRNA (Pfizer), first	relapse	• Oedema • Fatigue • AKI	3	Prednisolone	Complete remission after 4 weeks treatment
Holzworth 2021 USA	case 1 63 F	Hypertension, Tobacco dependence	No	NA	mRNA (Moderna), first	new	• Oedema • Dyspnoea • Fatigue • Frothy urine • Elevated blood pressure • Mild AKI	less than 7 days	Methylprednisolone, Prednisolone, Valsartan, Loop diuretic	NA
Komaba 2021 Japan	case 1 60s M	No	MCD	NA	mRNA (Pfizer), first	relapse	• Frothy urine	8	Prednisolone, Cyclosporine	Proteinuria resolved within 2 weeks treatment
D’Agati 2021 USA	case 1 77 M	Type 2 diabetes mellitus, Coronary artery disease, Obesity	No	NA	mRNA (Pfizer), first	new	• Oedema • Weight gain • Elevated blood pressure • Proteinuria	7	Methylprednisolone, Prednisolone, Furosemide, Bumetanide	No improvement after 3 weeks treatment
Maas 2021 Netherlands	case 1 80s M	Venous thromboembolism	No	NA	mRNA (Pfizer), first	new	• Oedema • Weight gain	7	Prednisolsteone	Improvement after 10 days treatment

ACEi: angiotensin converting enzyme inhibitor, AKI: acute kidney injury, F: female, GVHD: graft versus host disease, M: male, MCD: Minimal Change Disease, NA: not available

We identified a total of 94 cases of MCD following COVID-19 vaccination, of which one case was reported twice after relapsing following the second dose.

Fifty-five participants were males (59.1%, 55/93) and 38 (40.9%, 38/93) were females with a mean age of 45.02 years (SD:20.95). From the included patients 50 (50/94, 53.1%) were characterised as new-onset and 44 (46.9%, 44/94) as relapse. The mean age of individuals with MCD relapse was 41.6 (SD:20). For most of the patients (79.5%, 74/93) data regarding COVID-19 infection before or at the time of diagnosis was not provided. Among the remaining patients only 2 were previously infected with SARS-CoV-2. In 2 cases, vaccine brand was not reported (2.1%, 2/94). The majority of the patients received COMIRNATY (58.5%, 55/94), followed by COVID-19 Vaccine Moderna (20.2%, 19/94) and VAXZEVRIA (14%, 13/94), while 4 participants received COVID-19 Vaccine Janssen (3.2%, 3/94) and CoronaVac (1%, 1/94). In one case vaccine type was reported as modRNA (1%, 1/94). The majority of patients developed symptoms after the first dose (55.5%, 52/94), followed by the second dose (39.3%, 37/94), third dose (2.2%, 2/94), booster (1%, 1/94), both first and second doses (1%, 1/94), while in one case relevant data was not provided (1%, 1/94).

On average, the symptoms developed 16.68 days (SD: 22.85) after the administration of the vaccine irrespective of the dose. Data about symptomatology was reported in 68 individuals with the most common symptom being oedema (80.8%, 55/68), weight gain (26.5%, 18/68) and hypertension (16.1%, 11/68). MCD was confirmed with biopsy in 76 cases (80.8%, 76/94). Sixteen cases (17%, 16/94) were relapses and biopsy was not repeated. In two cases (2.1%), diagnosis was based on clinical suspicion (S1 Table). The majority of patients received steroids (91.5%, 86/94), while some patients were treated with immunosuppressive agents (22.3%, 21/94) and diuretics (17%, 16/94). More than half went to remission (61%, 57/94), while 18 achieved recovery or improved following treatment (19.1%, 18/94). Two people relapsed after treatment (2.1%, 2/94) and two cases (2.1%, 2/94) were reported as not recovered. In 15 cases (16%, 15/94) data about outcome was not provided.

### Quality of the studies

The mean quality score indicated that the studies reported on average 10 of the recommended 12 elements, defined by the guidelines. Ten studies had a perfect score of 12 while the second most common score was 11 (S2 Table).

## Discussion

The administration of COVID-19 vaccines has not been deemed as a causative factor for kidney disease. However, recent findings, primarily derived from case reports and case series, indicate that various kidney disorders such as Minimal Change Disease (MCD), IgA nephropathy, membranous glomerulopathy, and IgG4-related disease have been observed to initially manifest or relapse subsequent to SARS-CoV-2 vaccination. These observations suggest a potential link between COVID-19 vaccination and the occurrence or recurrence of MCD. In this study, we conducted a thorough screening of the existing literature to present a comprehensive summary of documented cases of MCD following SARS-CoV-2 vaccination. Our systematic review identified 46 relevant reports, involving a total of 93 patients, in which MCD was observed subsequent to the administration of various COVID-19 vaccines. In the majority of cases, symptoms began to emerge following the first vaccine dose, and clinical improvement was reported for most patients.

### Results in the context of the literature

MCD consists the most frequent cause of nephrotic syndrome in childhood and rarely affects adults. MCD generally presents with a sudden onset of symptoms and signs of nephrotic syndrome and requires histologic confirmation in adults. Its pathogenesis remains to be elucidated, however, evidence points towards T cell dysfunction being a major mechanism [66]. It has been previously proposed that a glomerular permeability factor is produced, attacks the glomerular filtration barrier and leads to the destruction of podocytes and subsequent proteinuria. It’s most commonly idiopathic, but infections, medications, vaccinations, malignancies, and allergens are among the secondary etiologic factors [67]. Infections including syphilis, hepatitis C and tuberculosis, and vaccinations against hepatitis B, influenza, measles and rubella are established triggering factors for the relapse of primary glomerulonephritis, potentially with a similar mechanism involved in the development of MCD [68].

In animal models the prevalence of CD8+ suppressor T-cells and subsequent cytokine-induced injury has been observed in the active phase of MCD [60, 69]. This could provide a possible explanation for the aforementioned cases since the existent vaccinations against COVID-19 are known to strongly induce T-cell activation and this could lead to immune mediated podocyte damage. It’s worth noting that during the vaccine-induced T-cell activation, interferon gamma and inerleukin-2 (IL-2) are increased and IL-2 has been found to be raised in the acute phase and relapses of idiopathic nephrotic syndrome [22]. Direct podocyte injury could also be implicated in MCD in both COVID-19 infection and vaccination and interestingly ACE-2 is expressed in podocytes, however there is currently not adequate evidence to establish a causative mechanism. Moreover, similarities between vaccine adjuvants and human proteins could lead to immune cross-reactivity and drug-induced hypersensitivity reactions through molecular mimicry [70, 71].

Even though MCD most commonly presents during childhood, it has been reported mainly in adults following COVID-19 vaccination, however this is to be expected considering the high vaccination rates in these age groups. MCD symptomatology commenced within 3 weeks from the first dose in more than half of the patients, although a significant amount of people developed symptoms after the second dose, which could be associated to the amplitude of the immune response. Symptoms did not differ from those commonly reported in literature and glucocorticoids were chosen as first-line treatment in 91.5% of the cases. Concerns about potential interference of immunosuppressive agents such as rituximab in the vaccination efficacy has been raised, however, relevant treatment to achieve best clinical response should be prioritised over immunisation in these cases. Overall the vast majority responded to treatment and maintained positive outcomes.

### Strengths and limitations

Our study represents the first systematic review conducted on the relationship between COVID-19 vaccination and the occurrence or relapse of MCD. Our findings present a comprehensive overview of published reports with quality assessment of the included studies.

However, it is important to highlight certain limitations linked to our study. One major limitation stems from the low quality nature of the case reports and case series included in this review, which can impact the validity and generalizability of the conclusions. These studies are susceptible to potential biases including overinterpretation and selection bias. Consequently, while the reported findings are interesting, they may not necessarily provide an accurate representation of the true effect of COVID-19 vaccination in relation to renal dysfunction. Therefore, establishing causality requires insight from mechanistic studies and well-designed appropriately powered prospective studies.

## Conclusion

While the current COVID-19 vaccines are generally considered safe and the advantages of vaccination outweigh the potential occurrence of adverse events, it is possible for patients to develop mild to moderate side effects, including complications related to renal dysfunction. Minimal change disease is possibly a condition clinical doctors and other healthcare professionals may expect to see in patients receiving COVID-19 vaccines. Although this adverse event is uncommon, considering the limited published data and the absence of confirmed causality, increased clinical awareness is crucial for the early recognition and optimal management of these patients.

## Supporting information

S1 Table Laboratory results and imaging findings for the included cases. (DOCX)

S2 Table Quality assessment of the included studies. (DOCX)

S1 Fig Prisma checklist. (DOC)
