# Supplementary material for: Minimal change disease following COVID-19 vaccination: A systematic review
Source: PLoS One. 2024 Mar 5;19(3):e0297568. doi: 10.1371/journal.pone.0297568 (PMC10914286; doi:10.1371/journal.pone.0297568)
Supplement: S1 Table — (DOCX) [file pone.0297568.s001.docx]

**Table S1.** Laboratory results and imaging findings for the included cases.

| **Author,**  **Year,**  **Country** | **Case number** | **Laboratory tests** | **Imaging** | **Biopsy report** |
| --- | --- | --- | --- | --- |
| Marampudi, 2022, USA | case 1 | - Urea: - Creatinine: 0.55mg/dL - Urine protein: 9977 mg/day [<300mg/day] - Antibodies: | N/A | N/A |
| Pella, 2022, Greece | case 1 | - Urea: 30 mg/dL - Creatinine: 0.99 mg/dL - Urine protein: 23.4g/day - Antibodies: | Abdominal CT: Ascites  Chest Radiograph: Unilateral Pleural effusion | No significant abnormalities, consistent with Minimal Change Disease |
| Alhosaini, 2022, UAE | case 1 | - Urea: - Creatinine: 0.85 mg/dL - Urine protein: 3+ - Antibodies: - Albumin: 1.5 g/dL - Urine Protein Creatinine Ratio: 5.6 g/g | Kidney Ultrasound: Increased echogenicity. Ascites and pleural effusion | Diffuse foot process effacements |
| Mochizuki, 2022, Japan | case 1 | - Urea: 15.8 mg/dL - Creatinine: 0.79 mg/dL - Urine protein: - Urine Protein Creatinine Ratio 7.07 g/g - Antibodies: | Chest Radiograph: Small pleural effusion.  Abdominal Ultrasound: Kidneys Normal, No urinary tract obstruction | Tubular epithelium congested and swollen  Sclerotic changes in small vasculature  Loss of foot process observed locally  No electron-dense deposits  Findings consistent with MCD |
| Park, 2022, Korea | case 1 | - Urea: - Creatinine: 5.38 mg/dL - Albumin: 1.4 g/dL - Urine protein: 16.1 g/day - Antibodies: | n/a | Biopsy consistent with MCD |
|  | case 2 | - Urea: - Creatinine: 0.72 mg/dL - Albumin: 2.1 g/dL - Urine protein: 16.1 g/day - Antibodies: | n/a | Biopsy feature consistent with MCD |
| Hartley, 2022, United Kingdom | case 1 | - Urea: 3+ protein - Creatinine: 310 mmol/L - Albumin: creatinine ratio: - Urine protein: 18.2 g/day - Antibodies: | Chest Radiograph: normal  Transthoracic echocardiography: normal | The kidney biopsy was consistent with MCD along with acute interstitial nephritis. |
|  | case 2 | - Urea: - Creatinine: 108 mmol/L - Albumin: 26 mmol/L - Albumin: Creatinine Ratio 800.7/mg/mmol - Urine protein: - Antibodies: | N/A | N/A |
| Leong, 2021, Singapore | case 1 | - Urea: - Creatinine: 54 µmol/L - Serum Albumin: 40 g/L - Urine protein: 4.23 g/day - Urine protein creatinine ratio: 413 mg/mmol - Antibodies: | n/a | n/a |
|  | case 2 | - Urea: - Creatinine: 64 µmol/L - Albumin: 17g/L - Urine protein: 0.75 g/day - Antibodies: | n/a | n/a |
| Tanaka, 2021, Japan | case 1 | - Urea: - Creatinine: 0.65 mg/dL - Serum Albumin: 1.5 g/dL - Urine protein : creatine ratio: 8.08g/gCr - Urine protein: - Antibodies: | n/a | Diffuse effacement of podocyte foot process with rouleau formation - suggestive of MCD |
| Jongvilaikasem, 2022, Thailand | case 1 | - Urea: - Creatinine: 2 mg/dL - Albumin: 2g/dL - Urine protein: 4+ - Antibodies: | n/a | Diffuse foot process effacement |
| Marinaki, 2021, Greece | case 1 | - Urea: - Creatinine: - Urine protein: 8.6 g/day - Antibodies: | n/a | Mild tubular atrophy and interstitial fibrosis  Diffuse foot process effacement with microvillous transformation |
| Biradar, 2021, India | case 1 | - Urea: - Creatinine: 0.72 g/dL - Albumin: 2.33 g/dL - Urine protein: 4+ - Urine : protein creatinine - Antibodies: | CXR: Normal  Kidney Ultrasound: normal shape, no evidence of obstruction | Globally sclerosed. Immunofluorescence negative. Consistent with MCD. |
| Unver, 2021, Turkey | case 1 | - Urea: - Creatinine: 0.6 mg/dL - Albumin: 2.2 g/dL - Urine protein: - Antibodies: | n/a | Consistent with MCD |
| Lebedev, 2021, Israel | case 1 | - Urea: 1.93g/dL - Creatinine: 2.3 mg/dL - Urine protein: - Antibodies: | CXR: Bilateral pleural effusions.  CT KUB: Normal kidney size, no obstruction | Mild congestion in glomerular capillaries. Extensive acute tubular injury, diffuse flattening of the podocytes. |
| Hanna, 2021, Canada | case 1 | - Urea: 116 µmol/L - Creatinine: - Urine protein: 24 g/L - Antibodies: | n/a | Acute tubular injurious changes, focal mild tubular atrophy, and interstitial fibrosis. Diffuse podocyte foot process effacement. Findings consistent with MCD |
| Baskaran, 2022, Australia | case 1 | - Urea: - Creatinine: 66 µmol/L - Albumin: 5 g/L - Urine protein: creatine ratio: 1484 mg/mmol - Antibodies: | n/a | Consistent with MCD with no evidence of immune deposits |
|  | case 2 | - Urea: - Creatinine: 636.66 µmol/L - Serum albumin 18 g/L - Urine protein: creatine 1631 mg/mmol - Antibodies: | n/a | Acute tubular injury with active interstitial inflammation and diffuse effacement of foot processes |
| Thappy, 2021, Qatar | case 1 | - Urea: - Creatinine: 80 μmol/L - Urine protein: 15g/24h - Albumin: 8g/L - Antibodies: | CXR: Prominent vascular markings and mild bilateral pleural effusion. Abdominal Ultrasound: normal sized kidneys | Normal glomeruli, 2+ mesangial deposition of immunoglobulin A (IgA), trace immunoglobulin G (IgG) and 1+ C3  Findings consistent with MCD |
| Abdulgayoom, 2021, Qatar | case 1 | - Urea: 5.8 mmol/L - Creatinine: 80 µmol/L - Albumin: 15 g/L - Urine protein: - Antibodies: | CXR: bilateral pleural effusion. Echo: normal ejection fraction, 62%. Abdominal Ultrasound: Kidneys - normal size, outline, and echotexture | No Immunoglobulin deposits on glomeruli |
| Klomjit, 2021, USA | case 6 | - Urea: - Creatinine: 2.18 g/dL - Serum albumin: 20 g/dL - Urine protein: 18g/day - Antibodies: | n/a | n/a |
|  | case 9 | - Urea: - Creatinine: 1.6 g/dL - Albumin: 2.5 g/dL - Urine protein: 19g/day - Antibodies: | n/a | n/a |
| Lim, 2021, Korea | case 1 | - Urea: - Creatinine: 1.13 mg/dL - Albumin: 1.6 g/dL - Urine protein: 8.6 g/day - Antibodies: | CT Abdomen: kidneys normal size and shape | Diffuse effacement of podocyte foot processes. |
| Salem, 2021, USA | case 1 | - Urea: - Creatinine: - Albumin: 2.3 g/dL - Urine protein: creatinine ratio: 6.4g/g - Antibodies: | n/a | Features consistent with MCD |
|  | case 2 | - Urea: - Creatinine: - Albumin 2.6 g/dL - Urine protein: creatine ratio 14.4 g/g - Antibodies: | n/a | Features consistent with MCD |
|  | case 3 | - Urea: - Creatinine: - Albumin: 2.8 g/dL - Urine protein: creatinine ratio 12.9 g/g - Antibodies: | n/a | Features consistent with MCD |
| Morlidge, 2021, UK | case 1 | - Urea: - Creatinine: 82 μmol/L - Albumin: 47 g/L - Urine protein: creatine ratio 213 mg/mmol - Antibodies: | n/a | n/a |
|  | case 2 | - Urea: - Creatinine: - Urine protein: 3+ protein dipstick - Antibodies: | n/a | n/a |
| Özkan, 2022, Turkey | case 1 | - BUN: 7 mg/dL - Creatinine: 0.57 mg/dL - Albumin: 3. 09 g/dL - Urine protein: 6034 mg/day - Antibodies: | n/a | n/a |
| Kervella, 2021, France | case 1 | - Urine protein: creatine ratio 2.4g/g | n/a | n/a |
| Chandra, 2022, USA | case 1 | - Urea: - Creatinine: 0.6 mg/dL - Albumin: 2.8 g/dL - Urine protein: 4.1 g/day - Antibodies: ANCA -ve, anti-dsDNA, ANA -ve, C3 -ve, C4 -ve | Renal Ultrasound showed normal-sized kidneys with no significant abnormalities. | Some glomeruli exhibited mild and focal mesangial expansion, no evidence of endocapillary proliferation.  Podocyte foot processes were diffusely effaced." |
|  | case 2 | - Urea: - Creatinine: 0.81 mg/dL - Albumin: 2.0 g/dL - Urine protein: creatinine ratio : 9.1 g/g - Antibodies: ANA -ve, ANCA -ve, C3 -ve, C4 -ve, amti – GBM -ve, HeP B -ve, Hep C -ve, | n/a | No glomerular abnormalities, no extensive foot process effacement.  Consistent with MCD since the biopsy was done as patient’s proteinuria was resolving. |
|  | case 3 | - Urea: - Creatinine: 1.8 mg/dL - Albumin: 1.5 g/dL - Urine protein: 1000 mg/dL - Urine protein : 5.2 g/day - Antibodies: | normal-sized kidneys with regular shape without hydronephrosis.  CXR: bilateral pleural effusion. | Interstitium showed small lymphocytic infiltrates, and there was acute tubular injury.  Mild glomerular deposits of IgM (+).  Diffuse podocyte foot process effacement. Findings consistent with MCD and acute tubular injury. |
|  | case 4 | - Urea: NA - Creatinine: 1.8 mg/dL - Albumin: 2.5 g/dL - Urine protein: creatinine ratio: 20 g/g - Antibodies: NA | NA | Podocyte, tubular epithelial, and endothelial injury suggestive of GVHD. EM showed extensive effacement of the podocyte foot processes consistent with MCD. |
| Hummel, 2022, France | case 1 | - Urea: NA - Creatinine: 10 mg/L - Albumin: 26 g/L - Urine protein: 5.2 g/g - Antibodies: Negative | NA | Consistent with MCD |
|  | case 3 | - Urea: NA - Creatinine: 11 mg/L - Albumin: 36 g/L - Urine protein: 1.4 g/day - Antibodies: Negative | NA | Consistent with MCD |
|  | case 4 | - Urea: NA - Creatinine: 6.3 mg/L - Albumin: 24.5 g/L - Urine protein: 7 g/g - Antibodies: NA | NA | Consistent with MCD |
|  | case 5 | - Urea: NA - Creatinine: 8.2 mg/L - Albumin: 27 g/L - Urine protein: 3.9 g/g - Antibodies: NA | NA | Consistent with MCD |
|  | case 6 | - Urea: NA - Creatinine: 6.1 mg/L - Albumin: 37 g/L - Urine protein: 6.1 mg/L - Antibodies: Negative | NA | Consistent with MCD |
|  | case 7 | - Urea: NA - Creatinine: 8.6 mg/L - Albumin: 37 g/L - Urine protein: 3 g/g - Antibodies: NA | NA | Consistent with MCD |
|  |  | NA | NA | Consistent with MCD |
|  | case 8 | - Urea: NA - Creatinine: 8.2 mg/L - Albumin: 37 g/L - Urine protein: 1.6 g/g - Antibodies: NA | NA | Consistent with MCD |
|  | case 9 | - Urea: NA - Creatinine: NA - Albumin: 28 g/L - Urine protein: 6.6 g/g - Antibodies: NA | NA | Consistent with MCD |
|  | case 10 | - Urea: NA - Creatinine: 6 mg/L - Albumin: 42 g/L - Urine protein: 6 g/g - Antibodies: NA | NA | Consistent with MCD |
|  | case 11 | - Urea: NA - Creatinine: 19 mg/L - Albumin: 16 g/L - Urine protein: 8.5 g/g - Antibodies: NA | NA | Consistent with MCD |
|  | case 12 | - Urea: NA - Creatinine: 13 mg/L - Albumin: 27 g/L - Urine protein: 8 g/g - Antibodies: NA | NA | Consistent with MCD |
|  | case 14 | - Urea: NA - Creatinine: 17 mg/L - Albumin: 20 g/L - Urine protein: 3.5 g/g - Antibodies: NA | NA | Consistent with MCD |
|  | case 15 | - Urea: NA - Creatinine: 9 mg/L - Albumin: 32 g/L - Urine protein: 3.6 g/g - Antibodies: NA | NA | Consistent with MCD |
|  | case 16 | - Urea: NA - Creatinine: 8 mg/L - Albumin: 43 g/L - Urine protein: 7.2 g/g - Antibodies: NA | NA | Consistent with MCD |
|  | case 17 | - Urea: NA - Creatinine: 9.3 mg/L - Albumin: 32 g/L - Urine protein: 2.8 g/g - Antibodies: NA | NA | Consistent with MCD |
|  | case 18 | - Urea: NA - Creatinine: 9.4 mg/L - Albumin: NA - Urine protein: 4.6 g/g - Antibodies: | NA | Consistent with MCD |
|  | case 19 | - Urea: NA - Creatinine: 9 mg/L - Albumin: 12 g/L - Urine protein: 5 g/g - Antibodies: NA | NA | Consistent with MCD |
|  | case 20 | - Urea: NA - Creatinine: 7.2 mg/L - Albumin: 19 g/L - Urine protein: 7.2 g/g - Antibodies: NA | NA | None |
|  | case 21 | - Urea: NA - Creatinine: 12.7 mg/L - Albumin: 13 g/L - Urine protein: 7.3 g/g - Antibodies: NA | NA | Consistent with MCD |
|  | case 22 | - Urea: NA - Creatinine: 13.7 mg/L - Albumin: 23 g/L - Urine protein: 3.3 g/g - Antibodies: NA | NA | Consistent with MCD |
|  | case 23 | - Urea: NA - Creatinine: NA - Albumin: NA - Urine protein: 10.3 g/g - Antibodies: NA | NA | Consistent with MCD |
|  |  | NA | NA | Consistent with MCD |
|  | case 24 | - Urea: NA - Creatinine: NA - Albumin: NA - Urine protein: 4.9 g/g - Antibodies: NA | NA | Consistent with MCD |
|  | case 25 | - Urea: NA - Creatinine: 23.8 mg/L - Albumin: 22 g/L - Urine protein: 10.1 g/g - Antibodies: NA | NA | Consistent with MCD |
| Güngör, 2022, Turkey | case 1 | - Urea: 5 mmol/L - Creatinine: 44.2 umol/L - Albumin: 12 g/L - Urine protein/creatinine ratio: 8.7 mg/mg - Antibodies: NA | NA | NA |
|  | case 2 | - Urea: 5 mmol/L - Creatinine: 42.4 umol/L - Albumin: 23 g/L - Urine protein/creatinine ratio: 4.1 mg/mg - Antibodies: NA | NA | NA |
| Fenoglio, 2022, Italy | case 5 | - Urea: NA - Creatinine: NA - Albumin: NA - Urine protein: NA - Antibodies: NA | NA | NA |
|  | case 7 | - Urea: NA - Creatinine: NA - Albumin: NA - Urine protein: NA - Antibodies: NA | NA | NA |
|  | case 8 | - Urea: NA - Creatinine: NA - Albumin: NA - Urine protein: NA - Antibodies: NA | NA | NA |
|  | case 12 | - Urea: NA - Creatinine: NA - Albumin: NA - Urine protein: NA - Antibodies: NA | NA | NA |
|  | case 16 | - Urea: NA - Creatinine: NA - Albumin: NA - Urine protein: NA - Antibodies: NA | NA | NA |
| Lim, 2022, Korea | case 2 | - Urea: NA - Creatinine: 1.54 mg/dL - Albumin: 1.6 g/dL - Urine protein: 8.6 g/day - Antibodies: None | NA | Diffuse foot process loss |
| Dormann, 2021, Germany | case 1 | - Urea: NA - Creatinine: NA - Albumin: NA - Urine protein: NA - Antibodies: NA | NA | NA |
|  | case 1 (2) | - Urea: NA - Creatinine: NA - Albumin: NA - Urine protein: NA - Antibodies: Negative | NA | Primary podocytopathy of the minimal change  Glomerulopathy (MCD) type |
|  | case 2 | - Urea: NA - Creatinine: NA - Albumin: Hypoalbuminemia - Urine protein: 22 g/g - Antibodies: NA | NA | Primary podocytopathy of the minimal change  Glomerulopathy (MCD) type |
| Anupama, 2021, India | case 1 | - Urea: NA - Creatinine: 1.09 mg/dL - Albumin: 2.15 g/dL - Urine protein: NA - Urine Protein Creatinine Ratio: 3.18 g/g - Antibodies: NA | NA | Glomerular walls did not stain for immunoglobulins or complement, but there was mesangial trapping of immunoglobulin M (IgM[1+]) and C3(1+) |
| Schwotzer, 2021, Switzerland | case 1 | - Urea: NA - Creatinine: 71 umol/L - Albumin: 23 g/L - Urine protein: 2+/3+ - Antibodies: NA | NA | NA |
| Hong, 2022, Taiwan | case 1 | - Urea: NA - Creatinine: 0.65 mg/dL - Albumin: 1.3 g/dL - Urine protein: 4+ - Antibodies: Negative | NA | Extensive podocyte effacement and microvilli transformation confirmed an MCD diagnosis. |
| Timmermans, 2022, Netherlands | case 1 | - Urea: NA - Creatinine: NA - Albumin: NA - Urine protein: NA - Antibodies: NA | NA | NA |
|  | case 2 | - Urea: NA - Creatinine: NA - Albumin: NA - Urine protein: NA - Antibodies: NA | NA | NA |
|  | case 3 | - Urea: NA - Creatinine: NA - Albumin: NA - Urine protein: NA - Antibodies: NA | NA | NA |
| Nakazawa, 2022, Japan | case 1 | - Urea: NA - Creatinine: 0.64 mg/dL - Albumin: 1.6 g/dL - Urinary Protein Creatinine Ratio: 7.71 g/g - Urine protein: 4+ - Antibodies: NA | Chest radiograph: bilateral pleural effusion  Abdominal ultrasonography: normal kidney size & edema of intestinal wall and ascites | NA |
| Arias, 2022, Spain | case 1 | - Urea: 33 mg/dL - Creatinine: 0.65 mg/dL - Albumin: 1.4 g/dL - Urine protein: - Antibodies: - MPO: 0.10 U/mL - PR3: 0.5 U/mL - Anti-GBM: negative | NA | Observing glomeruli optically normal with no immune deposits. |
| Haider, 2022, Italy | case 1 | - Urea: NA - Creatinine: Normal - Albumin: 3.0 g/dL - Urine protein: NA - Antibodies: NA | NA | NA |
| Fehr, 2021, Switzerland | case 1 | - Urea: NA - Creatinine: NA - Albumin: NA - Urine protein: NA - Antibodies: NA | NA | Minimal change disease with 90% loss of podocyte processes |
| Nagai, 2022, Japan | case 1 | - Urea: NA - Creatinine: NA - Albumin: NA - Urine protein: 14.4 g/day - Antibodies: - ANA: Negative | NA | NA |
| Caza, 2021, USA | case 3 | - Urea: NA - Creatinine: 2.2 g/dL - Albumin: 1.5 g/dL - Urine protein: 19.2 g/day - ANA: Negative - ANCA: NA | NA | MCD |
|  | case 4 | - Urea: NA - Creatinine: NA - Albumin: 1.9 g/dL - Urine protein: 10 g/day - Antibodies: - ANA: Negative - ANCA: Negative | NA | MCD |
|  | case 5 | - Urea: NA - Creatinine: 2.1 g/dL - Albumin: 1.9 g/dL - Urine protein: - Antibodies: - ANA: Positive - ANCA: Negative | NA | MCD |
|  | case 6 | - Urea: NA - Creatinine: - Albumin: 2.7 g/dL - Urine protein: - Antibodies: - ANA: Positive - ANACA: Negative | NA | MCD |
|  | case 7 | - Urea: NA - Creatinine: 0.7 g/dL - Urine protein: > 600 mg/dL - Albumin: 1.9 g/dL - Antibodies: - ANA: Negative - ANCA: Negative | NA | MCD |
|  | case 8 | - Urea: NA - Creatinine: 2.9 g/dL - Urine protein: 14 g/day - Albumin: NA - Antibodies: - ANA: Positive - ANCA: Negative | NA | MCD |
|  | case 9 | - Urea: NA - Creatinine: 0.86 mg/dL - Urine protein: 6 g/day - Albumin: NA - Antibodies: - ANA: Negative - ANCA: Negative | NA | MCD |
| Fornara, 2022, Italy | case 4 | - Urea: NA - Creatinine: 0.7 mg/dL - Albumin: NA - Urine protein: 4.1 g/day - Antibodies: NA | NA | NA |
| Leclerc, 2021, Canada | case 1 | - Urea: 103.8 mg/dL - Creatinine: 10.6 mg/dL - Albumin: 2.8 g/dL - Urine protein: NA - Urinary Protein Creatinine Ratio: 2321 mg/mmol - Antibodies: NA | Abdominal ultrasound: Normal  Computerized Tomography Thorax: Normal | Diffuse podocyte foot-process effacement (>80%) with focal microvillous transformation. |
| Mancianti, 2021, Italy | case 1 | - Urea: NA - Creatinine: 1.8 mg/dL - Albumin: 2.7 g/dL - Urine protein: 8 g/day - Antibodies: NA | Abdominal ultrasound: Unremarkable | Diagnostic for minimal change disease (MCD) |
| Holzworth, 2021, USA | case 1 | - Urea: NA - Creatinine: 1.48 mg/dL - Albumin: 0.7 g/dL - Urine protein: 13.4 g/day - Antibodies: NA | NA | 100% foot process effacement. |
| Komaba, 2021, Japan | case 1 | - Urea: NA - Creatinine: 0.99 mg/dL - Albumin: 2.8 g/dL - Urine protein: NA - Antibodies: NA | NA | NA |
| D'Agati, 2021, USA | case 1 | - Urea: NA - Creatinine: 2.33 mg/dL - Albumin: 2.5 g/dL - Urine protein: 23.2 g/day - Antibodies: NA | NA | 100% podocyte foot process effacement. |
| Maas, 2021, Netherlands | case 1 | - Urea: NA - Creatinine: 1.43 mg/dL - Albumin: 1.03 g/dL - Urine protein: 15.3 g/day - Antibodies: NA | NA | Diffuse podocyte foot-process effacement.  Findings consistent with MCD. |
