# Supplementary material for: Minimal change disease following COVID-19 vaccination: A systematic review
Source: PLoS One. 2024 Mar 5;19(3):e0297568. doi: 10.1371/journal.pone.0297568 (PMC10914286; doi:10.1371/journal.pone.0297568)
Supplement: S2 Table — (DOCX) [file pone.0297568.s002.docx]

**Table S2.** Quality assessment of the included studies.

| **Author, year** | **Q1** | **Q2** | **Q3** | **Q4** | **Q5** | **Q6** | **Q7** | **Q8** | **Q9** | **Q10** | **Q11** | **Q12** | **Overall** |
| --- | --- | --- | --- | --- | --- | --- | --- | --- | --- | --- | --- | --- | --- |
| Marampudi  2022 | ● | ● | ● | ● | ● | ● | ● | ● | ● | ○ | ● | ● | 11 |
| Pella  2022 | ● | ○ | ● | ● | ● | ● | ● | ● | ● | ● | ● | ● | 11 |
| Alhosaini  2022 | ● | ● | ● | ○ | ● | ● | ● | ● | ● | ● | ● | ● | 11 |
| Mochizuki  2022 | ● | ● | ● | ● | ● | ● | ● | ● | ● | ● | ● | ● | 12 |
| Park  2022 | ● | ● | ● | ○ | ○ | ● | ● | ● | ● | ○ | ○ | ● | 8 |
| Hartley  2022 | ● | ● | ● | ● | ● | ● | ● | ○ | ● | ● | ○ | ● | 10 |
| Leong  2021 | ● | ● | ● | ● | ● | ○ | ● | ○ | ● | ● | ● | ○ | 9 |
| Tanaka  2021 | ● | ● | ● | ● | ● | ● | ● | ● | ● | ● | ● | ● | 12 |
| Jongvilaikasem  2022 | ● | ● | ● | ● | ● | ● | ● | ● | ● | ● | ● | ● | 12 |
| Marinaki  2021 | ● | ● | ● | ○ | ● | ● | ● | ● | ● | ● | ● | ● | 11 |
| Biradar  2021 | ● | ● | ● | ● | ● | ● | ● | ● | ● | ● | ● | ● | 12 |
| Unver  2021 | ○ | ● | ● | ● | ● | ● | ● | ● | ● | ● | ○ | ● | 10 |
| Lebedev  2021 | ● | ● | ● | ● | ● | ● | ● | ● | ● | ○ | ○ | ● | 8 |
| Hanna  2021 | ● | ● | ● | ● | ● | ● | ● | ● | ● | ● | ● | ● | 12 |
| Baskaran  2022 | ● | ● | ● | ○ | ● | ● | ● | ● | ● | ○ | ● | ● | 10 |
| Thappy  2021 | ● | ● | ● | ● | ● | ● | ● | ● | ● | ● | ○ | ● | 11 |
| Abdulgayoom  2021 | ● | ● | ● | ● | ● | ● | ● | ● | ● | ○ | ● | ● | 11 |
| Klomjit  2021 | ○ | ● | ● | ○ | ● | ● | ● | ● | ● | ○ | ● | ● | 9 |
| Lim  2021 | ● | ● | ● | ● | ● | ● | ● | ○ | ● | ● | ● | ● | 11 |
| Salem  2021 | ● | ● | ● | ● | ● | ○ | ● | ● | ● | ○ | ○ | ○ | 8 |
| Morlidge  2021 | ● | ● | ● | ● | ○ | ○ | ● | ● | ● | ● | ○ | ○ | 8 |
| Özkan  2022 | ● | ○ | ● | ● | ● | ● | ● | ● | ● | ○ | ○ | ○ | 8 |
| Kervella  2021 | ● | ● | ● | ● | ● | ○ | ● | ● | ● | ○ | ○ | ○ | 8 |
| Chandra  2022 | ● | ● | ● | ● | ● | ● | ● | ● | ● | ● | ● | ● | 12 |
| Hummel  2021 | ○ | ● | ● | ○ | ● | ● | ● | ● | ● | ● | ● | ● | 10 |
| Güngör  2022 | ● | ● | ● | ○ | ● | ○ | ● | ● | ● | ○ | ○ | ● | 8 |
| Fenoglio  2022 | ○ | ● | ● | ○ | ○ | ● | ● | ● | ● | ○ | ● | ● | 8 |
| Lim  2022 | ○ | ● | ● | ● | ● | ● | ● | ● | ● | ○ | ● | ○ | 9 |
| Dormann  2021 | ● | ● | ● | ○ | ○ | ● | ● | ● | ● | ○ | ○ | ○ | 7 |
| Anupama  2021 | ● | ● | ● | ○ | ● | ● | ● | ● | ● | ○ | ○ | ○ | 8 |
| Schwotzer  2021 | ○ | ● | ● | ● | ● | ● | ● | ○ | ● | ● | ● | ○ | 9 |
| Hong  2022 | ○ | ● | ● | ● | ● | ● | ● | ● | ● | ● | ● | ● | 11 |
| Timmermans  2022 | ○ | ● | ● | ○ | ● | ○ | ● | ○ | ● | ○ | ○ | ● | 6 |
| Nakazawa  2022 | ● | ● | ● | ● | ● | ● | ● | ● | ● | ● | ● | ● | 12 |
| Arias  2022 | ● | ● | ● | ● | ● | ● | ● | ● | ● | ○ | ○ | ● | 10 |
| Haider  2022 | ● | ● | ● | ● | ● | ● | ● | ● | ● | ● | ● | ● | 12 |
| Fehr  2021 | ● | ● | ● | ● | ● | ● | ● | ● | ● | ○ | ○ | ● | 10 |
| Nagai  2022 | ● | ● | ● | ● | ● | ● | ● | ● | ● | ● | ○ | ● | ○ |
| Caza  2021 | ● | ● | ● | ○ | ● | ● | ● | ● | ○ | ○ | ● | ● | 9 |
| Fornara  2022 | ● | ● | ● | ● | ● | ● | ● | ● | ● | ○ | ○ | ○ | 9 |
| Leclerc  2021 | ● | ● | ● | ● | ● | ● | ● | ● | ● | ● | ● | ● | 12 |
| Mancianti  2021 | ● | ● | ● | ● | ● | ● | ● | ● | ● | ● | ● | ● | 12 |
| Holzworth  2021 | ● | ● | ● | ● | ● | ● | ● | ● | ● | ○ | ○ | ○ | 9 |
| Komaba  2021 | ● | ● | ● | ● | ● | ● | ● | ● | ● | ● | ○ | ○ | 10 |
| D’Agati  2021 | ● | ● | ● | ● | ● | ● | ● | ● | ● | ● | ○ | ● | 11 |
| Maas  2021 | ● | ● | ● | ○ | ● | ● | ● | ● | ● | ● | ○ | ○ | 9 |

Q1: Appropriate title ;Q2: Patient demographics ;Q3: Current health status ;Q4: Medical History ;Q5: Physical examination ;Q6: Patient disposition ;Q7: Drug Identification ;Q8: Dosage ;Q9: Administration of drug-reaction interface ;Q10: Concomitant therapies;Q11: Adverse events ;Q12: Discussion ● = 1; ○ = No;
