# Supplementary material for: Minimal change disease following COVID-19 vaccination: A systematic review
Source: PLoS One. 2024 Mar 5;19(3):e0297568. doi: 10.1371/journal.pone.0297568 (PMC10914286; doi:10.1371/journal.pone.0297568)
Supplement: Attachment — Submitted filename: response to reviewers.docx [file pone.0297568.s004.docx]

Imperial College London

Faculty of Medicine

Level 3, IRDB

Hammersmith Hospital Campus

Du Cane Road

London, UK

W12 0NN

konstantinos.kechagias18@imperial.ac.uk

12^th^ October 2023

To the Editor of “PLOS ONE’’

Re: Minimal change disease following COVID-19 vaccination: a systematic review

Dear Editor,

Thank you for considering our article ‘Minimal change disease following COVID-19 vaccination: a systematic review’ for publication in your journal.

We enclose below a point-by-point response to the reviewers’ comments and a revised version of the manuscript. We have also adjusted the format according to the journal’s guidance.

We look forward to your reply.

Yours sincerely

Konstantinos Kechagias MD, MSc

Clinical research fellow

Institute of Reproductive and Developmental Biology, Department of Metabolism Digestion and Reproduction, Imperial College London

**Reviewer #1: Please see the updated articles on various adverse effect of astrazeneca and other mrna covid vaccine. Authors missed the major complication such as sinus venous thrombosis as well as inflammatory cns disorders post vaccination and other relevant findings.
Also the information can be obtained from Medicines and Healthcare products Regulatory Agency (MHRA) data as well from Vaccine adverse events reporting system.**

Thank you for the comment. Although different types of adverse events secondary to Covid-19 vaccine have been recognized, the focus of the current literature review was minimal change disease. In the introduction section of the article, we briefly mention the plethora of other adverse events related to Covid-19 vaccines and we have now also included the MHRA guidance as reference.

Line 72.

**Reviewer #2: The research question is clear. The inclusion and exclusion criteria are well defined. The search strategy, study selection and data extraction appear comprehensive. Data synthesis is appropriate.
The interpretation of the findings and conclusion are accurate.**

**The following revisions are required:**

**Line 29 change “renal dysfunction” to more appropriate term – *Glomerular disease or chronic kidney disease. Line 45 change terms – “into remission “
Line 49 change “clinical doctors” to clinicians in the paper
Line 91 correct spelling of vaccine.**

We thank the reviewer for the annotations. The above have now been changed as per recommendations. Regarding line 91, the term vaccin* was used in order to identify articles including words such as vaccine, vaccines and vaccination.

Lines 29, 45 and 49.

**Line 147/8 biopsy reported in only 71% of cases (? diagnosis) – important limitation, if these were relapses then a second biopsy would be unlikely, although first time diagnosis would require a biopsy, specify if primary or relapse.**

We thank the reviewer for the comment. We elaborated on the use of biopsy as a diagnostic tool. Biopsy reports were provided for 67 cases (71.2%, 67/94) and were consistent with MCD. MCD was confirmed with biopsy in 76 cases (80.8%, 76/94). Sixteen cases (17%, 16/94) were a relapse and biopsy was not repeated. In two cases (2.1%), diagnosis was based on clinical suspicion (Nagai et al, Nakazawa et al).

Lines 147 and 148.

**First line of discussion “renal dysfunction” term should be changed.**

Thank you for the annotation. Renal dysfunction has been replaced by kidney disease.

Line 161

**Second line of paragraph: Results in context of literature:
“Evidence points towards T cell dysfunction being a major Mechanism” Is this immune dysregulation with dysfunction of T regulatory cells as with other cases of MCD?**

We thank the reviewer for this comment. Yes, this refers to the dysfunction of T regulatory cells and the hypothesis that a glomerular permeability factor is produced that subsequently attacks the glomerular membrane, which has been proposed in the past as a potential mechanism for MCD. This has now been clarified in the manuscript.

Line 175

**Line 3-4 “glomerular capillary wall” could be restated as the** **glomerular filtration barrier.**
Thank you for the annotation. This has now been restated as suggested.

Lines 3 and 4.

**Last line “requires histologic confirmation in adults” is fundamental to diagnosis. MCD is a histological diagnosis. Unless there was relapse which did not warrant a biopsy, then all other cases would require confirmatory histology. If this is not so, then it should be listed as a limitation of the review.**

We thank the reviewer for the comment, and we agree. Biopsy reports were provided for 67 cases (71.2%, 67/94) and were consistent with MCD. MCD was confirmed with biopsy in 76 cases (80.8%, 76/94). Sixteen cases (17%, 16/94) were relapses and biopsy was not repeated. In two cases (2.1%), diagnosis was based on clinical suspicion.

Lines 147-149

**Reviewer #3: It is a relevant topic with billions of doses of vaccines used in such a short period of time and also Being RNA based.**

**1. Need clarity on that only 76% had histological classification of their nephrosis. what criteria were used to establish minimal change.**

We thank the reviewer for the comment and we agree. Biopsy reports were provided for 67 cases (71.2%, 67/94) and were consistent with MCD. MCD was confirmed with biopsy in 76 cases (80.8%, 76/94). Sixteen cases (17%, 16/94) were a relapse and biopsy was not repeated. In two cases (2.1%), diagnosis was based on clinical suspicion (Nagai, Nakazawa).

Lines 147-149

**2. Was there any particular vaccine type that caused the most or least number of nephrotic syndrome cases. I know that then we have to look at the number of vaccine doses but was there a particular type involved with the new cases compared to the relapses?**

We thank the reviewer for the comment. In the results section, both the vaccine type and the number of doses before the development of nephrotic syndrome are mentioned. The majority of the patients received COMIRNATY (58.5%, 55/94), followed by COVID-19 Vaccine Moderna (20.2%, 19/94) and VAXZEVRIA (14%, 13/94), while 4 participants received COVID-19 Vaccine Janssen (3.2%, 3/94) and CoronaVac (1%, 1/94). In one case vaccine type was reported as modRNA (1%, 1/94). The majority of patients developed symptoms after the first dose (55.5%, 52/94), followed by the second dose (39.3%, 37/94), third dose (2.2%, 2/94), booster (1%, 1/94), both first and second doses (1%, 1/94), while in one case relevant data was not provided (1%, 1/94).

Lines 138-143

**3. There are 2 diabetics with a possibility of diabetic nephropathy? Was minimal change confirmed on histology? The other secondary involvement were cardiac problems such as hypertension, hyperlipidaemia, and arrthytmias** **although hypertension and hyperlipdaemis can be caused by the nephrosis**

Thank you for your comment. Patients with the above comorbidities had histological diagnosis of MCD.

**5. If only 76% had renal histology, which criteria were used to prove minimal change?**

We thank the reviewer for this comment. Biopsy reports were provided for 67 cases (71.2%, 67/94) and were consistent with MCD. MCD was confirmed with biopsy in 76 cases (80.8%, 76/94). Sixteen cases (17%, 16/94) were relapses and biopsy was not repeated. In two cases (2.1%), diagnosis was based on clinical suspicion.

Lines 147-149

**6. Infections that were mentioned in the discussion such as Hep 'B' cause membranous nephropathy.**

We thank the reviewer for this comment. We mentioned Hep B and other viruses as triggering factors for primary glomerulonephrosis including MCD given that a similar pathophysiological mechanism could be implicated.
